# Effects of estrogen deficiency during puberty on maxillary and mandibular growth and associated gene expression – an μCT study on rats

**DOI:** 10.1186/s13005-021-00265-3

**Published:** 2021-04-22

**Authors:** Erika Calvano Küchler, Rafaela Mariana de Lara, Marjorie Ayumi Omori, Guido Marañón-Vásquez, Flares Baratto-Filho, Paulo Nelson-Filho, Maria Bernadete Sasso Stuani, Moritz Blanck-Lubarsch, Agnes Schroeder, Peter Proff, Christian Kirschneck

**Affiliations:** 1 Department of Orthodontics, University of Regensburg, Franz-Josef-Strauss-Allee 11, 93053 Regensburg, Germany; 2 Department of Pediatric Dentistry, School of Dentistry of Ribeirão Preto, University of São Paulo, Avenida do Café, Ribeirão Preto, SP 14040-904 Brazil; 3 Private Practice, Curitiba, PR 80060-000 Brazil; 4 School of Dentistry, Univille University, R. Paulo Malschitzki, Joinville, SC 89219-710 Brazil; 5 Department of Orthodontics, University of Muenster, Albert-Schweitzer-Campus 1, Building W30, 48149 Münster, Germany

**Keywords:** Maxilla, Mandible, Tooth, Estrogen, Gene expression

## Abstract

**Background:**

Estrogen is a well-known and important hormone involved in skeletal homeostasis, which regulates genes involved in bone biology. Some studies support that estrogen is important for craniofacial growth and development. Therefore this in vivo animal study aimed to investigate, whether and in which way low estrogen levels in the prepubertal period affect craniofacial development in the postpubertal stage and to quantify the gene expression of RANK, RANKL and OPG in cranial growth sites in ovariectomized estrogen-deficient rats during puberty.

**Methods:**

Control (sham-operated, *n* = 18) and ovariectomy (OVX, n = 18) surgeries were performed on 21-days-old female Wistar rats. Animals euthanized at an age of 45 days (pubertal stage) were used for gene expression analyses (*n* = 6 per group) and immunohistochemistry of RANK, RANKL and OPG. Animals euthanized at 63 days of age (post-pubertal stage) were used for craniofacial two-dimensional and three-dimensional craniofacial measurements using μCT imaging (*n* = 12 per group).

**Results:**

In the μCT analysis of the mandible and maxilla many statistically significant differences between sham-operated and OVX groups were observed, such as increased maxillary and mandibular bone length in OVX animals (*p* < 0.05). Condylar volume was also significantly different between groups (p < 0.05). The sham-operated group showed a higher level of RANK expression in the midpalatal suture (*p* = 0.036) and the RANKL:OPG ratio levels were higher in the OVX group (*p* = 0.015).

**Conclusions:**

Our results suggest that estrogen deficiency during the prepubertal period is associated with alterations in the maxillary and mandibular bone length and condylar growth.

## Background

Postnatal craniofacial skeletogenesis is a unique process, in which many factors can affect growth and development. Estrogen is an important hormone involved in the skeletal homeostasis that regulates different aspects of bone metabolism, development, modeling and remodeling. Endogenous levels of estrogen change according to age and gender [1]. During puberty, there is an increase in estrogen levels leading to the development of secondary sexual characteristics and a significant increase in growth rate [2]. During the pubertal growth spurt, this hormone plays an important role controlling growth plate acceleration and fusion [3]. Estrogen deficiency has been reported in syndromes and genetic conditions [4–6], menstrual disorders [7], primary ovarian insufficiencies [8], underweight [9], excessive exercise [10] and chemotherapy [11]. It is well known that estrogen deficiency can cause osteoporosis [12, 13], decrease mineral density in bones and delay the epiphyseal maturation [14].

It is well established that sex steroids, including estrogen, regulate the RANK (receptor activator of nuclear factor-κB), RANKL (receptor activator of nuclear factor-κB ligand) and OPG (osteoprotegerin) axis, members of the tumor-necrosis-factor superfamily. One of the most important downstream mediators of the action of estrogen on bone is RANKL [15], which is crucial for osteoclast differentiation, activation and survival. OPG is a soluble decoy receptor binding RANKL, inhibiting osteoclastogenesis via the RANK receptor on osteoclast precursor cells [16, 17]. RANKL, which is expressed membrane-bound by osteoblasts and can also be released in soluble form, acts via its receptor RANK, which is expressed on the cell membrane of osteoclasts and osteoclast precursor cells [18]. RANK, RANKL and OPG are essential, non-redundant factors for osteoclast biology [15].

Some studies using rodent animal models were conducted previously to describe, how estrogen affects growth and development of craniofacial structures [19–23] and a variety of outcomes were observed. Some studies demonstrated a growth inhibition of the craniofacial complex in estrogen-deficient newborn mice [14, 20, 21], while increased condyle dimensions were observed, when the estrogen deficiency was induced in 8-weeks-old mice [19]. In a study performed on rats with estrogen deficiency induced at an age of 30 days, no alterations in craniofacial growth were observed [1]. On the other hand, in our previous study with estrogen deficiency induced in the prepubertal stage (age: 21 days), estrogen-deficient animals presented increased maxillary and mandibular measurements [23]. In this previous project from our research group, the two-dimensional radiographic analysis performed suggested that estrogen deficiency from the prepubertal stage affects the dimensions of the maxilla and mandible in female rats [23]. Therefore, in the present study, we used high-resolution micro-computed tomography (μCT) to analyze the maxillary and mandibular skeletal dimensions of adult female rats, which were subjected to estrogen deficiency during the prepubertal stage, in three dimensions. We also evaluated, if estrogen deficiency affects craniofacial growth via the RANK, RANKL and OPG axis by investigating their expression at growth sites of both maxilla and mandible during puberty and estrogen deficiency.

## Materials and methods

### Ethical aspects

The present study was performed and reported in accordance with the ARRIVE guidelines [24]. The Ethical Committee in Animal Experimentation from the School of Dentistry of Ribeirão Preto, University of São Paulo, Brazil, approved the protocol of this study (2014.1.721.58.7).

### Sample selection

The sample size was calculated for the application of independent-measures t tests based on estimates from a pilot study in radiographic images (*n* = 5). Several calculations were performed considering the results on each comparison related to the measures evaluated in the morphometric analysis and gene expression analysis. The highest estimations of the outcomes analyzed were considered as the sample size required. The following parameters were considered for the highest sample size estimation for the morphometric analysis: effect size = 1.4, α = 0.05 (5% error), power = 0.8, number of groups = 2. Regarding the gene expression outcome, the effect size considered was 2.2 and the other parameters were the same to the above-mentioned. The calculation estimated a minimum total sample of 20 animals for morphometric analysis and 10 animals for gene expression (total *n* = 30). Considering the possibility of using non-parametric statistics and possible losses, an increase of 20% was performed, which resulted in the adjustment of the sample size for 36 animals (6 rats for gene expression and 12 rats for morphometric analysis, per group). The sample size calculation was performed in G*Power (version 3.1.9.7). Posteriorly, the animals were randomly assigned into OVX and sham-operated control groups using sealed envelopes to ensure the allocation concealment.

The housing room was temperature and humidity controlled and rats had ad libitum access to food. Briefly, the rats were anesthetized using an intraperitoneal injection of 10% ketamine hydrochloride (55 mg/kg of gross body weight) and 2% xylazine hydrochloride (10 mg/kg of gross body weight). At an age of 21 days (prepubertal stage), bilateral ovariectomy was performed in the OVX group and placebo surgery was performed in the sham-operated group according to the protocol of Omori et al. [23]. At an age of 45 days (pubertal stage) animals were euthanized for gene expression analyses. At an age of 63 days (post-pubertal stage - young adult) the remaining rats were euthanized for morphometric μCT analyses. Ages/developmental stages were established according to Sengupta [25].

As previously described by Chen et al. [26] and Omori et al. [23], body weight and uterus atrophy were significantly higher in the OVX rats than in sham-operated rats at 63 days of age (*p* < 0.05), confirming the success of ovariectomy. In case that the uterus atrophy was not observed, the animal would be excluded from the analysis.

### Morphometric μCT analysis

The entire maxilla and mandible of each rat was retrieved after euthanasia and scanned with the micro-CT system phoenix v|tome|x s 240/180 research edition from GE Sensing & Inspection Technologies GmbH in the Regensburg Center of Biomedical Engineering. Scanning parameters for the rats’ upper jaws were as follows: 60 kV voltage, 800 μA current, 333 ms time, 1500 images, voxel size 55 μm, fast scan; while for the rats’ lower jaws settings were as follows: 60 kV voltage, 600 μA current, 333 ms time, 1500 images, voxel size 37 μm, fast scan. Reconstructed volumes were processed using the corresponding manufacturer’s software phoenix datos|× 2 reconstruction 2.4.0 (GE Sensing & Inspection Technologies GmbH, Wunstorf, Germany).

**Fig. 1:**
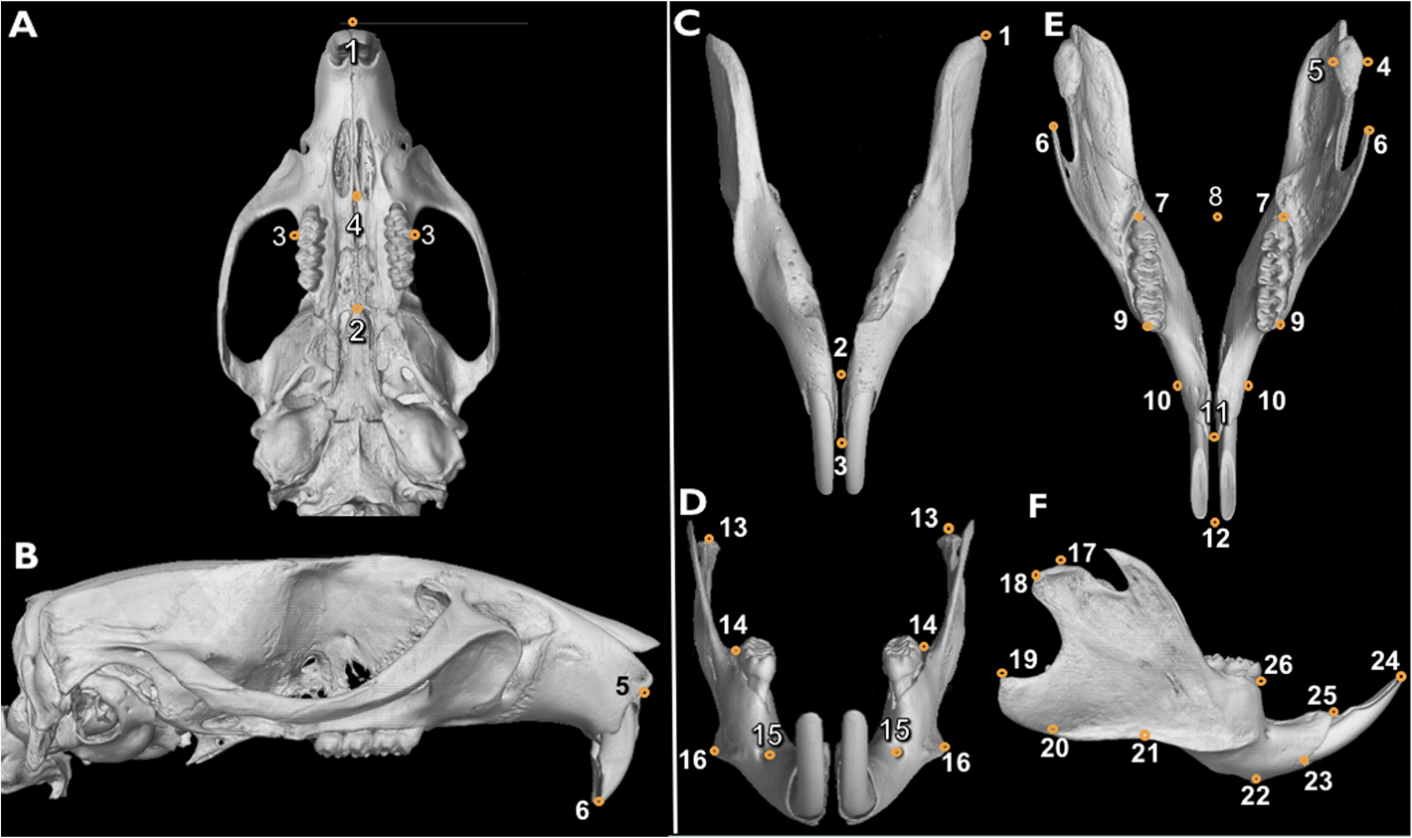
Landmarks used for maxillary and mandibular measurements. **a** Inferior view of maxilla. **b** Lateral view of skull. **c** Inferior view of mandible. **d** Frontal view of mandible. **e** Superior view of mandible. **f** Lateral view of mandible

The 3D data of the maxilla and mandible were both analyzed using VGSTUDIO MAX 3.3 – Voxel Data Analysis and Visualization (Volume Graphics GmbH, Heidelberg, Germany) and Image J software (National Institutes of Health, Bethesda, MD, USA). To perform the maxillary and mandibular morphometric measurements, the 3D jaw images obtained by μCT were aligned in dorsal, lateral and ventral view and standardized scaled 2D images were taken for all rats. Image J software was used to measure linear (mm) and angular (°) dimensions within these 2D images. The used landmarks for both maxilla and mandible are demonstrated in Fig. 1 and described in Table 1 and were selected based on Wei et al. [27], Fujita et al. [20], Corte et .al [28], Wang et al. [29] and Perilo et al. [30]. The linear and angular measurements evaluated here are described in Table 2. One single calibrated examiner blindly performed all morphometric analyses. Each measurement was taken three times and the mean of the three measurements was used to perform statistical analyses. To assess intra-examiner reliability, the Intra-class Correlation Coefficient (ICC) was calculated to assess the concordance of measurements.

**Table 1:** Description of maxillary and mandibular landmarks used

***Landmarks***	***Description***
**Maxilla**	
1	Intersection of nasal bones
2	Posterior point of the suture between palatines and the anterior border of the mesopterygoid fossa
3	The most prominent lateral point on the buccal surface of the upper first molar
4	Anterior limit of upper first molar
5	Anterior notch on zygomatic process
6	Anterior most point of alveolus upper incisor
7	The most prominent point between the incisal edges of the incisors
**Mandible**	
1	Most posterior point of mandibular body base
2	Lowest point of alveolar bone around lower incisor
3	Highest point of alveolar bone around lower incisor
4	Lateral face of condylar process
5	Medial face of condylar process
6	Top of coronoid process
7	Distal face of third lower molar
8	Posterior limit of lower molars
9	Vestibular face of mesiobuccal cusp of first lower molar
10	Lateral face of both sides of mentum bone
11	Highest point of alveolar bone around lower incisor
12	Most prominent point between incisal edges of lower incisor
13	The topmost point of condylar process
14	The most prominent lateral point on the buccal surface of the lower first molar
15	Mental foramen
16	The point located at the gonial angle of the mandible
17	Most superior point of the condyle
18	Posterior-most point of condyle
19	Tip of mandibular angle
20	Point on most inferior contour of angular process of mandible
21	Point in deepest part of antegonial notch curvature
22	Inferior point on mandibular symphysis
23	Inferior rim point on lower incisor alveolus
24	Most prominent point between incisal edges of lower incisor
25	Superior rim point on lower incisor alveolus
26	Point on intersection between the mandibular alveolar bone and mesial surface of first molar

**Table 2:** Linear and angular measurements evaluated in this study

***Landmarks***	***Description***	***References***
**Maxilla**		
1–2	Maxillary arch length	–
3–3	Maxillary intermolar distance	–
1–4	Maxillary diastema length	–
2–4	Maxillary posterior segment length	–
6–7	Maxillary central incisor length	–
**Mandible**		
18–19	Mandibular height – from most posterior point of condyle to most posterior point of mandiblular angle	Wei et al.*,* 2017
18–25	Upper mandibular length	Wei et al.*,* 2017
19–22	Lower mandibular length	Wei et al.*,* 2017
20–22	Mandibular plane length	Wei et al.*,* 2017; Fujita et al.*,* 2004
17–22	Diagonal mandibular length	Wei et al.*,* 2017; Fujita et al.*,* 2004
17–19	Distance between the most superior point of the condyle to mandible angle	Wei et al.*,* 2017; Fujita et al.*,* 2004
23–24	Mandibular central incisor length	Wei et al.*,* 2017; Fujita et al.*,* 2004
25–26	Distance between first lower molar mesial face to lower central incisor buccal face	Wei et al.*,* 2017; Fujita et al.*,* 2004
17–20	Mandibular height- from most superior point of the condyle to mandible base	Wei et al.*,* 2017
17–19–20-22	Mandibular angle composed between the lines 17–19 and 20–22	Fujita et al.*,* 2004
20–22-24	Mandibular angle composed by the landmarkers 20, 22 and 24	Fujita et al.*,* 2004
17–20-22	Mandibular angle composed by the landmarkers 17, 20 and 22	Fujita et al.*,* 2004
7–7	Mandibular arch width	Corte et al.*,* 2019
9–9	Mandibular intermolar distance	Corte et al.*,* 2019
8–12	Mandibular arch length	Corte et al.*,* 2019
8–9	Mandibular posterior segment length	Corte et al.*,* 2019
9–11	Mandibular diastema length	Corte et al.*,* 2019
10–10	Mandibular interdiastemal breadth	Corte et al.*,* 2019
6–6	Intercoronoidal breadth	Corte et al.*,* 2019
4–5	Thickness of condylar process	Wang et al.*,* 2016
1–2	Inferior mandibular body length	Corte et al.*,* 2019
2–3	Anterior mentum height	Corte et al.*,* 2019
13–13	Mandibular superior third width	Wei et al.*,* 2017
14–14	Mandibular middle third width	Perilo et al.*,* 2014
16–16	Mandibular inferior third width	Perilo et al.*,* 2014
15–15	Distance between mentum foramina	Corte et al.*,* 2019

**Fig. 2:**
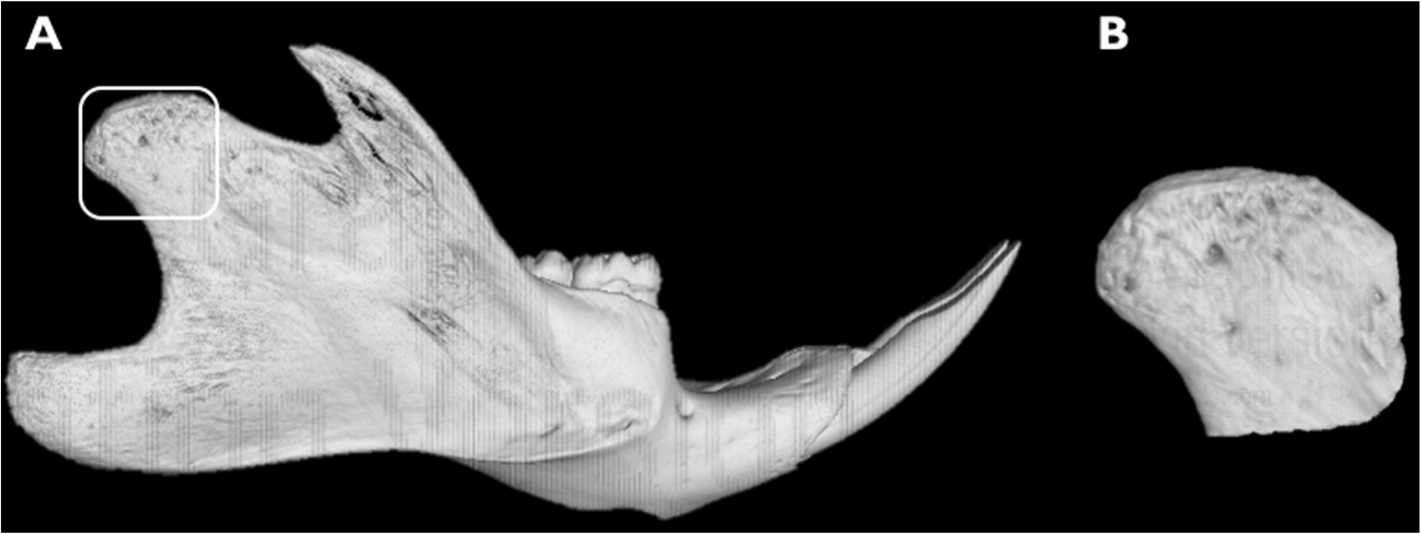
μCT imaging example illustrating the mandible. **a** Pre-sized square in the condyle. **b** Region of interest

Condylar volume was also obtained using VGSTUDIO MAX 3.3. All mandibles were aligned in a lateral view of the right condyle, which was separated from the mandible using a specific software tool to measure its volume (mm^3^). The amount of bone selected to measure the volume was standardized by positioning a pre-sized square (12 mm × 12 mm) over the condyle, in a way that the superior edge of the square matched with the most superior border of the condyle, and the left edge of the square matched with the most posterior border of the condyle in order to select the region of interest (ROI) (Fig. 2 a and b).

### Gene expression of RANK, RANKL and OPG

Gene expression analysis was performed in growth sites of the maxilla (midpalatal suture) and mandible (condyle, mandibular angle, symphysis/parasymphysis and coronoid process) at the pubertal stage. Bone samples were dissected after euthanasia at the age of 45 days and stored in RNAlater (Life Technologies Corporation – Carlsbad, CA, USA) at − 80 °C until processing. Total RNA was extracted using the mirVana™ miRNA Isolation kit (Ambion/Life Technologies™, USA). Complementary DNA (cDNA) was synthesized by reverse-transcription with a High Capacity kit (Applied Biosystems, Foster City, CA, USA).

Quantitative real-time polymerase chain reaction (RT-qPCR) was blindly performed using a StepOnePlus™ sequence detection system (Applied Biosystems™, Foster City, CA, USA). The thermal cycling was carried out by starting with a hold cycle of 95 °C for 20 min, followed by 40 amplification cycles of 95 °C for 1 min and 60 °C for 20 min. Pre-designed TaqMan® primers and probes (Thermo Fisher Scientific, MA, USA) for RANK (Rn 04340164-m1), RANKL (Rn 00589289-m1 RankL) and OPG (Rn 00563499-m1 OPG). GAPDH (Rn01462661-g1) and ACTB (Rn01412977-g1) were used as endogenous controls and confirmed to be stably expressed. The relative levels of mRNA expression were determined by the 2^-∆∆^CT method. GAPDH and ACTB genes were used for sample normalization according to Livak and Schmittgen [31] to calculate relative gene expression. All procedures were performed following the respective manufacturer’s instructions and according to established protocols.

### Immunohistochemical analysis of RANK, RANKL and OPG

Immunohistochemical analysis was performed as previously described [32]. The slides were incubated over night at 4 °C with the primary antibodies diluted in 1% BSA: anti-RANK (polyclonal rabbit antibody H300 sc:9072; diluted 1:25; Santa Cruz Biotechnology Inc., Santa Cruz, CA, USA), anti-RANKL (polyclonal goat antibody sc:7628; diluted 1:25; Santa Cruz Biotechnology Inc., Santa Cruz, CA, USA), and anti-OPG (polyclonal goat antibody n-20 sc:8468; diluted 1:25; Santa Cruz Biotechnology Inc., Santa Cruz, CA, USA). Later, after being washed, the slides were incubated with a biotinylated secondary antibody (goat anti-rabbit IgG-B sc-2040 and rabbit anti-goat IgG-B sc-2774; Santa Cruz Biotechnology Inc., diluted 1:200) for 1 h at room temperature. The streptavidinbiotin-peroxidase complex (ABC kit, Vectastain; Vector Laboratories Inc., Burlingame, CA, USA) was subsequently added for 30 min, followed by the addition of chromogen 3,3′ diaminobenzidine tetrahydrochloride hydrate (DAB; Sigma-Aldrich Corp., St. Louis, MO, USA) with 3% hydrogen peroxide in PBS for 1 min. Finally, the slides were counterstained with Harris’ hematoxylin. The identification of RANK, RANKL and OPG was performed at a magnification of 20x under conventional light using a Olympus BX-BX61 microscope (Olympus, Tokio, Japan) equipped with a camera connected to a computer (DELL®, Dell Inc., Round Rock, USA) and the software DP2-BSW® (Olympus, Tokio, Japan). The results were expressed qualitatively, according to the presence/absence of immunostaining in the regions of interest.

### Statistical analysis

Sample normality was analyzed by Shapiro-Wilk tests. The comparative analysis was performed by Student’s t tests to assess differences in morphometric measurements between OVX and sham-operated groups. For gene expression analyses Mann-Whitney U tests were used. Statistical significance was set at *p* ≤ 0.05. All analyses were performed using the Prism 8 software (Graph Pad Software Inc., San Diego, California, USA).

## Results

Intraexaminer reliability of measurements was good with ICC ≥ 0.78. Eight animals died during the experiment. All the remaining animals were included, as follow: For gene expression analysis, samples from 5 OVX rats and samples from 4 sham-operated rats were used. For morphometric analysis, 8 skulls of OVX rats and 11 skulls of sham-operated rats were evaluated.

The overall dimensions in millimeter (mm) of the reconstructed μCT images of the rats’ maxilla and mandible are presented in Table 3. The maxillary posterior segment length was smaller in the OVX group (*p* = 0.012), while the maxillary central incisor length was bigger in the OVX group (*p* = 0.010). In the mandibular sagittal plane, mandibular height (*p* = 0.006), upper mandibular length (*p* = 0.037), mandibular plane length (*p* = 0.019), diagonal mandibular length (*p* = 0.014), the distance between the condyle to mandibular angle (*p* = 0.025) and mandibular height (*p* = 0.004) were bigger in the OVX group. Also, the mandibular angle composed by the lines intersecting the landmarks 17–19 and 20–22 was higher in the OVX group (*p* = 0.009). In the mandibular transversal plane, the mandibular interdiastemal breadth (*p* = 0.002) and the thickness of the condylar process (*p* < 0.0001) were larger in the OVX group.

**Table 3:** Means and standard deviations of 2D and 3D measurements

Measurements	Groups		***p*** Value
	OVX Mean (SD)	Sham-operated Mean (SD)	
**Maxilla**			
Arch length (1–2)	30.21 (0.67)	30.53 (1.26)	0.533
Intermolar distance (3–3)	9.77 (0.39)	9.87 (0.50)	0.672
Diastema length (1–4)	17.56 (0.50)	17.50 (0.93)	0.858
Posterior segment length (2–4)	9.78 (0.38)	10.36 (0.47)	0.012*
Maxillary central incisor length (6–7)	11.11 (0.23)	10.50 (0.60)	0.010*
**Mandible**			
Mandibular height (18–19)	10.96 (0.66)	10.05 (0.61)	0.006*
Upper mandibular length (18–25)	35.47 (1.75)	33.55 (1.88)	0.037*
Lower mandibular length (19–22)	29.90 (1.81)	28.25 (1.76)	0.063
Mandibular plane length (20–22)	23.04 (1.40)	21.26 (1.52)	0.019*
Diagonal mandibular length (17–22)	29.93 (1.12)	28.15 (1.56)	0.014*
Distance between the condyle to mandibular angle (17–19)	14.35 (0.52)	14.07 (0.46)	0.025*
Mandibular central incisor length (23–24)	12.67 (1.03)	13.22 (0.52)	0.142
Distance between first molar to central incisor (25–26)	8.72 (0.35)	8.41 (0.60)	0.213
Mandibular height (17–20)	17.66 (1.09)	16.16 (0.90)	0.004*
Angle composed between the lines 17–19 and 20–22	114.21 (2.71)	109.88 (3.77)	0.009*
Angle composed by the landmarks 20, 22 and 24	135.33 (1.62)	134.49 (1.75)	0.305
Angle composed by the landmarks 17, 20 and 22	99.47 (5.9)	100.21 (2.44)	0.599
Arch width (7–7)	12.40 (0.94)	12.28 (0.76)	0.748
Intermolar distance (9–9)	11.39 (1.10)	11.15 (0.57)	0.543
Arch length (8–12)	25.72 (2.06)	25.71 (0.89)	0.988
Posterior segment length (8–9)	10.13 (0.86)	10.02 (0.43)	0.725
Diastema length (9–11)	9.35 (1.25)	8.99 (0.33)	0.365
Mandibular interdiastemal breadth (10–10)	6.00 (0.32)	5.49 (0.28)	0.002*
Intercoronoidal breadth (6–6)	27.20 (2.08)	26.62 (2.03)	0.558
Thickness of condylar process (4–5)	2.29 (0.15)	1.94 (0.12)	<0.0001*
Inferior mandibular body length (1–2)	39.01 (0.96)	38.23 (2.43)	0.405
Anterior mentum height (2–3)	5.63 (0.68)	5.42 (0.59)	0.479
Mandibular superior third width (13–13)	19.69 (1.03)	19.95 (1.47)	0.670
Mandibular middle third width (14–14)	14.60 (0.75)	14.86 (1.19)	0.595
Mandibular inferior third width (16–16)	18.18 (1.06)	17.77 (1.24)	0.466
Distance between mentum foramens (15–15)	10.32 (0.68)	10.38 (0.84)	0.873

*indicates statistically significant difference (p < 0.05). All measurements are given in millimeter (mm)

The condyle volume (mm^3^) was significantly increased in the OVX group (mean = 10.10; SD = 0.54) compared to the sham-operated group (mean = 8.91; SD = 1.15) (*p* = 0.016).

Gene expression of RANK, RANKL, OPG and the RANKL:OPG ratio are presented in Table 4. The sham-operated group had a higher level of RANK expression in the midpalatal suture (*p* = 0.036). The RANKL:OPG ratio was higher in the mandibular condyle of the OVX group (*p* = 0.015).

**Table 4:** Gene expression in the growth sites assessed

Growth sites	***Groups***	RANK		RANKL		OPG		RANKL:OPG	
		Mean (SD)	***p***-value	Mean (SD)	p-value	Mean (SD)	p-value	Mean (SD)	p-value
**Midpalatal suture**	***OVX***	0.40 (0.08)	0.036*	1.22 (0.54)	0.239	0.85 (0.18)	0.306	1.40 (0.96)	0.313
	***Sham-operated***	0.67 (0.02)		0.65 (0.05)		0.68 (0.06)		0.96 (0.16)	
**Condyle**	***OVX***	1.80 (1.48)	0.174	1.57 (0.84)	0.061	0.35 (0.13)	0.868	4.48 (1.61)	0.015*
	***Sham-operated***	0.44 (0.07)		0.40 (0.14)		0.37 (0.16)		1.17 (0.43)	
**Mandibular angle**	***OVX***	0.43 (0.13)	0.191	0.41 (0.03)	0.209	0.41 (0.11)	0.136	1.02 (0.19)	0.588
	***Sham-operated***	0.27 (0.16)		0.28 (0.18)		0.28 (0.10)		0.93 (0.24)	
**Symphysis / parasymphysis**	***OVX***	0.62 (0.56)	0.806	1.00 (0.56)	0.451	1.05 (0.36)	0.453	0.92 (0.40)	0.804
	***Sham-operated***	0.35 (0.43)		0.65 (0.06)		0.77 (0.44)		1.04 (0.68)	
**Coronoid process**	***OVX***	0.43 (0.14)	0.412	0.39 (0.14)	0.904	0.29 (0.09)	0.703	1.37 (0.34)	0.285
	***Sham-operated***	0.33 (0.09)		0.34 (0.11)		0.32 (0.06)		1.06 (0.33)	

Note: *means statistically significant difference (p < 0.05)

**Fig. 3:**
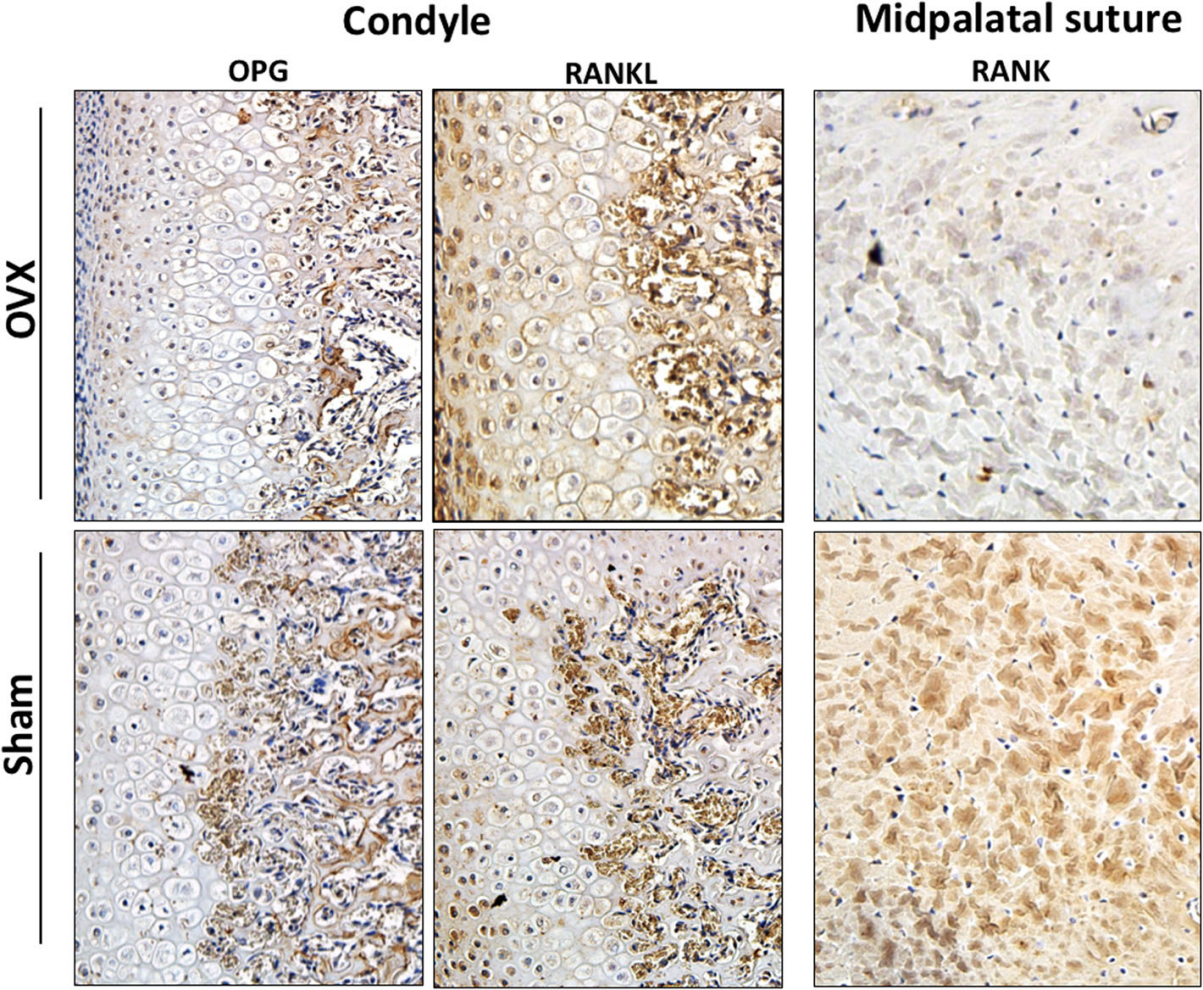
Representative photomicrographs of the immunostaining for RANK, RANKL and OPG of the condyle and the midpalatal suture at 45 days of age. Magnification: 20x

The immunohistochemical analysis showed a pattern of results similar to that observed in gene expression tests (Fig. 3). Immunostaining for RANK was more intense in the midpalatal suture chondrocytes for the sham-operated group. RANKL staining was more pronounced in the proliferative and hypertrophic layers of the mandibular condyle for the OVX group, while, on the contrary, OPG staining was more intense in the condyle for the sham-operated group.

## Discussion

Estrogen deficiency during (pre) pubertal stage may impact on the development of the facial complex. Our study using a rodent model suggested that estrogen is one of the factors involved in the maxillary and mandibular growth and development. Since low estrogen levels in women and teenage girls can be caused by different conditions and their effects depends on the individual’s age and general health, clinicians should be aware of the possible potential impact of estrogen deficiency in dental arch development of girls.

In animal models, it is well known that low levels of estrogen can lead to changes in bone microarchitecture in femurs and mandibles [33], osteoporosis [13] and alterations in the alveolar bone [34] as well as alterations in craniofacial development [14, 19–21, 23]. In our study, we were able to extend upon the radiographic two-dimensional cephalometric linear findings reported by Omori et al. [23], who found that estrogen deficiency during puberty led to alterations in maxillary and mandibular dimensions. The use of images from μCT in the present study allowed us to perform a more reliable and complete analysis, adding more landmarks and different view positions of both arches resulting in a more detailed description of the phenotypes. Radiographic cephalometric linear and angular analysis of murine skulls has been developed long ago [35] and is very similar to that used in humans, which has practical and successful clinical applications [27]. Although some landmarks are difficult to identify on two-dimensional radiographs of mouse and rat skulls, radiograph-based cephalometric has been used successfully to identify morphometric changes in estrogen models of mice [14, 20] and rats [23, 36]. However, μCT-based craniofacial measurements have the advantage of a high resolution and the ability to determine both morphology and volume.

In our study, the thickness of the condylar process, as well as condylar volume were bigger in the OVX group. Larger measurements of condyle breadth were found in mice, when estrogen deficiency was induced in eight-week-old mice [20], although a normal width, low trabecular bone volume of the condyle and reduction in bone mineral density were also found [12, 13, 21].

We were able to observe that although the posterior segment length of the maxilla was smaller in OVX group, the others measurements statistically different among the groups - maxillary dimension (maxillary central incisor length) and the mandibular dimensions - were bigger in the OVX group. The phenotype observed in the maxilla of OVX group, in which the posterior length was smaller, could reflect a maxillary retrusion in humans. On the other hand, in mandible, linear and angular measurements related to the mandibular height and mandibular length were bigger in OVX group. These growth patterns in humans could lead to a mandibular protrusion (in the sagittal plane) and also a brachyfacial biotype tendency.

An explanation for maxillary and mandibular differences could be the fact that estrogen performs two opposite functions in two distinct phases of puberty. During the prepubertal period, the activity of estrogen is systemic in accordance with growth hormone (GH) causing bone elongation [2]. After this period of growth comes the period known as postpubertal, when estrogen acts at the local level causing epiphyseal fusion, resulting in bone maturation [2, 33]. Estrogen is needed during bone growth and development for proper closure of epiphyseal growth plates both in females and in males. Also, within the young skeleton, estrogen deficiency leads to increased osteoclast formation and enhanced bone resorption [37]. It is important to emphasize that estrogen plays a role in bone via the RANK/RANKL/OPG triad.

Our gene expression results and immunohistochemical analysis demonstrated that estrogen deficiency during puberty might affect the expression of RANK, RANKL and OPG in the craniofacial growth sites. Mandibular condylar cartilage is known as the center of most pronounced growth in the mandible and the craniofacial complex, and is associated with morphogenesis of the craniofacial skeleton [38]. The RANKL:OPG ratio was higher in the condyle of the OVX group, which may indicate a higher bone remodeling activity in this group. However, this results should be interpreted with caution. Although gene expression analysis was performed in both groups at the same age, at 45 days-old OVX and sham-operated group could be in a different pubertal stages, once higher levels of estrogen accelerates puberty [2]. It is important to mention that the condylar cartilage is a heterogeneous tissue comprising cells at different stages of chondrogenic maturation. Condylar cartilage is designated as secondary cartilage and differs from primary cartilage in histological organization; modes of proliferation, differentiation and calcification [30]. Chondrocytes also express and produce RANK, RANKL and OPG [39, 40]. A study in a rodent model concluded that OPG plays a protective role in the postnatal survival of condylar chondrocytes [41]. A possible limitation of our study is that gene expression was evaluated only in one time-period, which not allowed us to evaluate how the levels of RANK, RANKL and OPG behave throughout craniofacial growth during puberty.

The midpalatal suture is an important growth site in the maxilla. RANK in the midpalatal suture was differentially expressed among groups. Mature osteoclasts have the RANK receptor for their activation. Once RANKL binds to RANK, this activates the process of bone resorption. Although we observed that RANK expression was higher in the sham-operated group, RANKL and OPG expression were not statistically different among the groups in the midpalatal suture. In order to start bone remodeling, RANKL should bind RANK [41]. Therefore, it is possible that the statistical difference observed here does not have a biological impact on bone remodeling.

Briefly, our results using an estrogen-deficient animal model support that estrogen is important for maxillary and mandibular development during puberty and could impact the expression of the RANK/RANKL/OPG system in growth sites of the facial complex. However, the role of high of estrogen levels (hyperestrogenism) in maxillary and mandibular growth, as well as the role of estrogen in contraceptives are still unknown. It is possible that an estrogen excess, which could occur when taking estrogen-based contraceptives during puberty, might have the opposite effect as detected in the OVX animals, that is an inhibiting effect on mandibular and maxillary growth as well as craniofacial growth in general. Further studies should elucidate the effect of high levels of estrogen during facial growth. The levels of RANK, RANKL and OPG in maxillary and mandibular growth sites during different periods of puberty should also be investigated.

## Conclusions

Estrogen deficiency during puberty could be involved on maxillary and mandibular dimensions and on RANK/RANKL/OPG expression at important growth sites of the jaws.

## Data Availability

The datasets used and/or analyzed during the current study are available from the corresponding author on reasonable request.

## Abbreviations

OVX: Ovariectomy
μCT: Microtomography
RANK: Receptor activator of nuclear factor-κB
RANKL: Receptor Activator of NF-κB Ligand
RANKL: Receptor Activator of NF-κB
OPG: Osteprogesterin
GAPDH: Glyceraldehyde-3-phosphate dehydrogenase
ACTB: Actin beta
BSA: Bovine serum albumin
PBS: Phosphate buffered saline
